# MDM2 Binding Protein Induces the Resistance of Hepatocellular Carcinoma Cells to Molecular Targeting Agents *via* Enhancing the Transcription Factor Activity of the Pregnane X Receptor

**DOI:** 10.3389/fonc.2021.715193

**Published:** 2021-06-24

**Authors:** Qiyu Jiang, Yan Ma, Jingjing Han, Jingdong Chu, Xuemei Ma, Lijun Shen, Bo Liu, Bo-an Li, Jun Hou, Qian Bi

**Affiliations:** ^1^ Institute of Infectious Disease, Department of Infectious Disease, The Fifth Medical Center of Chinese PLA General Hospital, Beijing, China; ^2^ Endoscopy Center, Department of Hepatology, The Fifth Medical Center of Chinese PLA General Hospital, Beijing, China; ^3^ Department of Gastroenterology and Hepatology, The First Medical Center of Chinese PLA General Hospital, Beijing, China; ^4^ Department of Gastroenterology, Sangzhi County National Hospital, Zhangjiajie City, China; ^5^ Department of Clinical Laboratory, The Fifth Medical Center of Chinese PLA General Hospital, Beijing, China

**Keywords:** MDM2 binding protein, Pregnane X receptor, hepatocellular carcinoma, molecular targeting agent, multi-drug resistance, drug metabolism and clearance

## Abstract

The MDM2 binding protein (MTBP) has been considered an important regulator of human malignancies. In this study, we demonstrate that the high level of MTBP’s endogenous expression is correlated with poor prognosis of advanced hepatocellular carcinoma (HCC) patients who received sorafenib. MTBP interacted with the Pregnane X receptor (PXR) and enhanced the transcription factor activity of PXR. Moreover, MTBP enhanced the accumulation of PXR in HCC cells’ nuclear and the recruitment of PXR to its downstream gene’s (*cyp3a4’s*) promoter region. Mechanically, the knockdown of MTBP in MHCC97-H cells with high levels of MTBP decelerated the clearance or metabolism of sorafenib in HCC cells and led to the resistance of HCC cells to sorafenib. Whereas overexpression of MTBP in in MHCC97-L cells with low levels of MTBP showed the opposite trend. By establishing the interaction between MTBP and PXR, our results indicate that MTBP could function as a co-activator of PXR and could be a promising therapeutic target to enhance the sensitivity of HCC cells to molecular targeting agents.

## Introduction

The hepatocellular carcinoma (HCC) has been a fatal threat to the health of the people of China due to the high rates of hepatitis and the resistance or insensitivity to HCC cells in relation to molecular targeting agents. This has been considered an important influencing factor for poor prognoses of patients with advanced HCC (1–4). The PXR (pregnane X receptor) is a member of the nuclear receptors super-family and mainly expresses in the liver and gastrointestinal tract tissues (5, 6). It has long been considered to be the regulatory center for the metabolism and detoxification of exogenous drugs and toxicants; results of recent studies show that it is also a key regulator for anti-tumor drug resistance in cancerous cells (7–10). Upon binding and activating by ligands, PXR can recruit the promoter or enhancer regions to mediate the transcription of its downstream drug-resistance-related genes, inducing *cyp3a4* and *abcb1* (also named multi-drug resistance 1 [*mdr-1*]/ATP binding cassette subfamily B member 1 [ABCB1]). It does this by encoding the P-glycoprotein [P-gp]) to mediate the elimination of sorafenib in cancerous cells *via* the N-oxide metabolism of sorafenib or by decreasing the intracellular accumulation of it, which in turn reduces the clinical efficacy or resistance of the drug during treatment (5–10).

The MDM2 binding protein (MTBP) has a molecular weight of 104 kDa and was originally found to interact with MDM2 (murine double minute 2) (11, 12). Recently, MTBP has been regarded as an important regulator of human cancer cells’ proliferation or metastasis (13, 14). MTBP could promote human cancer cells’ proliferation or metastasis by functioning as a co-activator of c-MYC (cellular-myelocytomatosis viral oncogene) or ZEB2 (Zinc Finger E-Box Binding Homeobox 2) (13). In this study, MTBP was found to function as a co-activator of PXR, further enhancing PXR’s downstream drug-resistance-related genes. MTBP was highly expressed in HCC clinical specimens compared to those of the paired non-tumor tissues. The knockdown of MTBP in MHCC97-H with high levels of endogenous MTBP could have decelerated the elimination or clearance of sorafenib and enhanced the antitumor effect of sorafenib in MHCC97-H cells. Whereas overexpression of MTBP in in MHCC97-L cells with low levels of MTBP showed the opposite trend.

## Materials and Methods

### Clinical Specimens and Agents

The use of clinical specimens and cell lines were approved by the ethics committee of the Fifth Medical Center, General Hospital of Chinese PLA (People’s Liberation Army). All methods and experiments were carried out in accordance with the Helsinki Declaration. A total number of 52 patients with advanced HCC received sorafenib as described in our previous publications (Feng et al. [2018] and Shao et al. [2018]) (7, 8). The cDNA samples were conserved at -80°C (7, 8). The expression of MTBP in the cDNA of the patients was examined by quantitative polymerase chain reaction (qPCR). The hepatic cell lines L-02, HepG2, MHCC97-H, MHCC97-L, BEL-7402, SMMC-7721 and Hu7 cells were conserved in our lab and cultured in Dulbecco’s Modified Eagle Medium (DMEM, Hyclone, Thermo Fisher Scientific Corporation, Waltham, MA, USA). We added Fetal Bovine Serum (FBS, Invitrogen, Thermo Fisher Scientific Corporation, Waltham, MA, USA) at 37°C in DMEM to culture cells. The antitumor agents (the molecular targeted agents), sorafenib, regorafenib, lenvatinib, apatinib and anlotinib, were chemically synthesized by Dr. and Prof. Shuang Cao in Wuhan Institute of Technology. Rifampicin, a typical agonist of PXR, and ketoconazole, a typical antagonist of PXR, were purchased from Selleck Corporation.

For cell-based experiments, the molecular targeting agents were dissolved in DMSO (dimethyl sulfoxide) and diluted with phenol red-free DMEM (Thermo Fisher Scientific Corporation) (15, 16). For the animal experiments, the formulation (oral liquid for the oral administration) of molecular targeting agents was prepared by using PEG400 and Twenn80 (17, 18). The lentivirus particles with the full length of MTBP and the siRNA of MTBP were purchased from the Vigene Corporation, Jinan City, Shandong Province, China.

### Luciferase Examination

The luciferase reporters of PXR (PXRE-Luc, XREM-Luc, DR3-Luc and ER6-Luc) were described in our previous publication (7, 8). The cells were cultured and suspended using the phenol red-free DMEM supplemented with 0.5% charcoal-stripped fetal bovine serum (FBS; Hyclone, Logan, Utah, USA). Cells were transfected with the MTBP and siMTBP and analyzed by luciferase activation or β-gal activation following the instructions provided in methods section of the manuscript by Yang et al., Zhao et al. and Cui et al. (19–21).

### Quantitative Polymerase Chain Reaction

The mRNA samples were extracted from the HCC cells and reverse-transcribed into the cDNA samples by use of an RNeasy Mini kit (Qiagen, Valencia, CA, USA) following the manufacturer’s instructions. The quantitative real-time PCR (qPCR) was performed following the methods described by Jia et al. and Yin et al. (22, 23). The level of β-actin mRNA was measured as a loading control. Primers used in the qPCR experiments included: (1) MTBP forward sequences 5′-TCCTGTAGTTTCGTCAGATCCT-3′ and reverse sequences 5′-CCGTTTCAATCGGGATACTTCA-3′; (2) ABCB1 forward sequences 5’-GCTG TCAAGGAAGCCAATGCCT-3’ and reverse sequences 5’-TGCAATGGCGATCCTCTGCTT C-3’; (3) CYP3A4 forward sequences 5’-CCGAGTGGATTTCCTTCAGCTG-3’ and reverse sequences 5’-TGCTCGTGGTTTCATAGCCAGC-3’; and (4) β-actin forward sequences 5’-CACCA TTGGCAATGAGCGGTTC-3’ and reverse sequences 5’-AGGTCTTTGCGGATGTCC ACGT-3’.

### Western Blot Experiments

Total protein samples were extracted from HCC cells and the subcutaneous tumor tissues; western blot experiments were performed following the methods described in our previous publication. The antibodies against P-GP (P-glycoprotein, encoded by *abcb1*), CYP3A4, Lamin A, PXR and β-Actin were purchased from Abcam Corporation, Cambridge, UK. The expression levels of proteins were examined by their antibodies. The blots were visualized *via* the chemiluminescence by use of an ECL kit (Amersham Biosciences, Piscataway, NJ, USA).

### Pharmacokinetic Examination

The sustaining of sorafenib in HCC cells and tumor tissues was examined by liquid chromatography–mass spectrometry/mass spectrometry (LC-MS/MS) methods to reveal the elimination or clearance of sorafenib in HCC cells. Pharmacokinetic experiments were performed according to the protocols described in our previous work (7, 8).

### ChIP Experiments

The HCC cells were transfected with the siRNA of MTBP and MTBP were treated with rifampicin for 30min. Then, cells were harvested for ChIP analysis and the experiments were performed following the methods described by Ma et al. (2016) and Wang et al. (2018) (24, 25). The primers to amply the promoter region (the PXRE region [−362/+52]) of the promoter of *cyp3a4* were (1) forward primer: 5’-AGATCTGTAGGTGTGGCTTGTTGG-3’; (2) reverse primer: 5’-TGTTG CTCTTTGCTGGGCTA TGTGC-3’; (3) Input (genome DNA sequence): forward primer: 5’-AA CCTATTAACTCACCCTTGT-3’; and (4) reverse primer: 5’-CCTCCATTCAAAAGATCTTATTATTTAG CATCTCCT-3’.

### The Subcellular Sub-Fraction Analysis

The HCC cells were transfected with the siRNA of MTBP and MTBP were treated with rifampicin for 30min to 1h. Next, the cells were harvested and the subcellular sub-fraction was performed following the methods provided by Yang et al. and Lu et al. (26, 27). Lamin A was used as the indicator of the nuclear sub-fraction and β-Actin was chosen as the indicator of the cytoplasm sub-fraction. The protein levels of MTBP, PXR, Lamin A and β-Actin were examined by their antibodies.

### Cell Survival Examination

The HCC cells were transfected with the siRNA of MTBP and MTBP were treated with the indicated concentration of molecular targeting agents (10μmol/L, 3μmol/L, 1μmol/L, 0.3μmol/L, 0.1μmol/L, 0.03μmol/L and 0.01μmol/L) for 48h. The cells were then analyzed by the MTT (3-[4,5-dimethyl-2-thiazolyl[-2,5-diphenyl-2-H-tetrazolium bromide, Thiazolyl Blue Tetrazolium Bromide) experiments following the methods descripted by Zhang et al. (28). The related survival cell number was determined by measuring the O.D. 490nm. The inhibitory rates of agents on HCC cells were calculated as [(the O.D. 490nm values of the control group) – (the O.D. 490nm values of the treatment group)]/(the O.D. 490nm values of control group) * 100%. The *IC_50_* values of agents on HCC cells were calculated based on these inhibitory rates (29, 30).

### The *In Vivo* Antitumor Effect of Molecular Targeting Agents on HCC Cells

The animal experiments were reviewed approved by the Institutional Animal Care and Use Committee, the Fifth Medical Center, Chinese PLA. All animal experiments were performed in accordance with the UK Animals (Scientific Procedures) Act, 1986 and the associated guidelines. Nude mice ages 4 to 6 weeks were purchased from the Si-Bei-Fu Corporation (Beijing, China). For the subcutaneous tumor model, HCC cells were cultured and harvested to prepare the single cell suspension. Then, the cell-suspension was injected into the subcutaneous positions of the mice (about 1×10^6^ cells for each injection point). Three to four days after injection, mice received the molecular targeting agents *via* oral administration once every two days. After three to four weeks’ treatment, the mice were collected and their tumor volumes were calculated as width^2^*length/2 (31, 32). Their tumor weights were measured with a precise balance. The inhibition rates of molecular targeting agents on HCC cells’ subcutaneous growth were calculated based on tumor weights and tumor volumes. For the intrahepatic tumor model, HCC cells were first injected into the mice to form tumor tissues (7, 8). Next, the tumors were collected to prepare micro-blocks of tumor tissues which were mixed with medical hydrogel to form hydrogel drips with tumor tissues. **Supplemental Table 1** shows the weights of the micro-blocks formed by the MHCC97-H cells and the MHCC97-L cells and transplanted into the mice. The hydrogel drips were adhered onto the surface of the liver organs of the mice. Three to four days after injection, the mice received the molecular targeting agents *via* oral administration once every two days. After three to four weeks’ treatment, mice received a micro-PET screening to examine the intrahepatic growth of HCC cells. After micro-PET screening, the mice were harvested and their livers with nodules formed by HCC cells were collected (7, 8). The results were shown as micro-PET images, the quantitative results of micro-PET, the images of liver organs with nodules or the quantitative results of liver organs’ images with nodules formed by HCC.

### The Statistical Analysis

Statistical significance analyses were analyzed by using the statistical software (software version: SPSS 9.0, the IBM corporation, Armonk, New York, USA). The IC50 values or the Statistical significance was analyzed by Bonferroni correction with or without two ways ANOVA.

## Results

### MTBP Enhanced the Transcription Factor Activation of PXR

**Figure 1:**
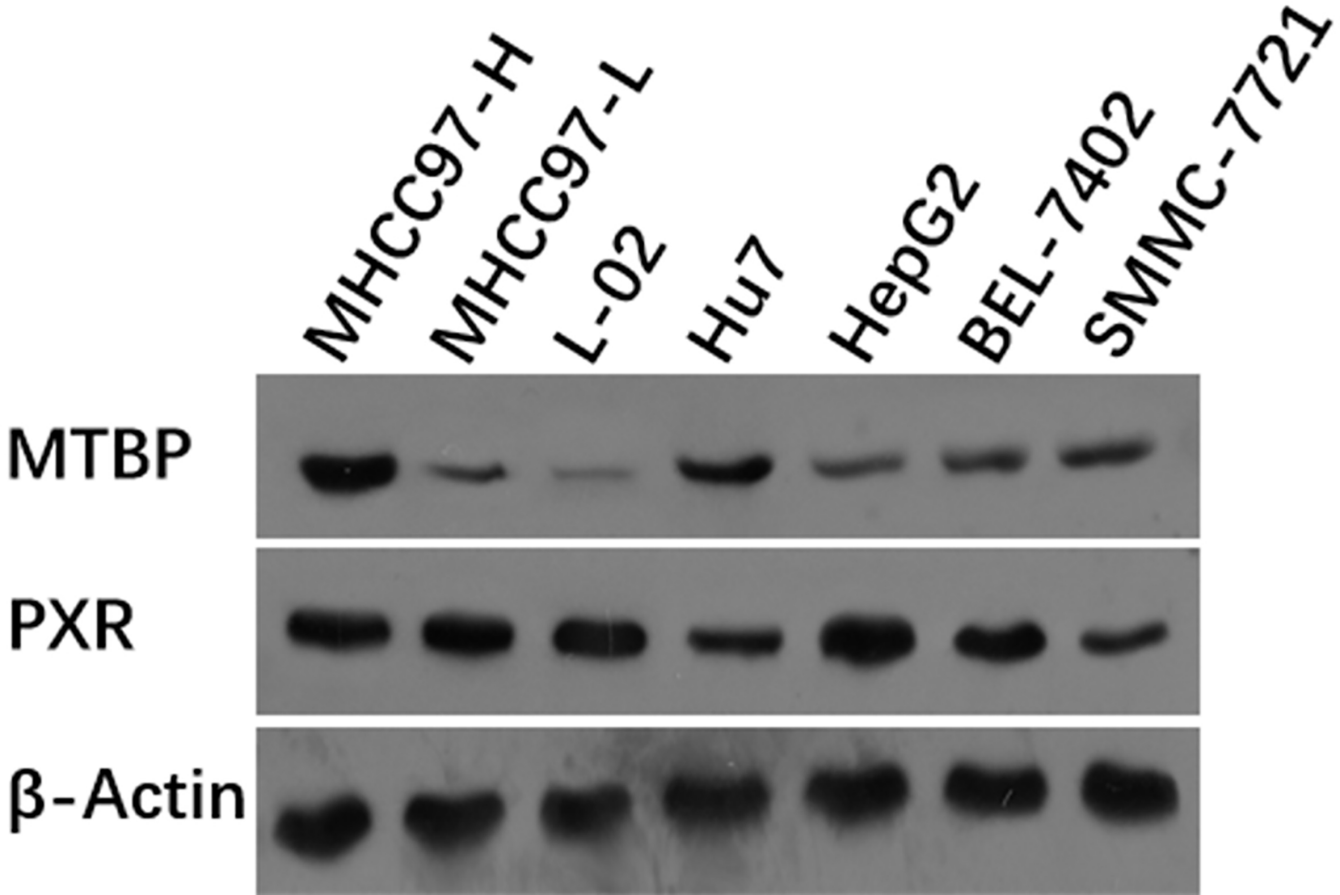
The expression of the highest level of MTBP among the hepatic cell lines. The hepatic cell lines MHCC97-H (a highly aggressive HCC cell line), MHCC97-L (a lowly aggressive HCC cell line), L-02 (a non-tumor hepatic cell line), Hu7 (a HCC cell line with dysfunction of P53), HepG2 (a HCC cell line), BEL-7402 (a HCC cell line) and SMMC-7721 (a HCC cell line) were cultured and harvested for western blot experiments. The protein levels of MTBP in these cells were examined by the antibodies. The β-Actin was chosen as the loading control.

First, the expression of MTBP in hepatic cells was examined. As shown in **Figure 1**, among hepatic cell lines, MHCC97-H, a highly aggressive HCC cell line, expressed the highest level of endogenous MTBP, whereas MHCC97-L, an HCC cell line with low aggressiveness, expressed the lowest level of endogenous MTBP. Therefore, MHCC97-H cells were used to knockdown MTBP’s expression and MHCC97-L cells were used to overexpress MTBP.

Next, the effect of MTBP on PXR’s transcription factor activation was examined. As shown in **Table 1**, rifampicin, a typical agonist of PXR, induced the transcription factor activation of PXR. The overexpression of MTBP enhanced the activation of PXR induced by rifampicin and the *EC_50_* values decreased; the knockdown of MTBP decreased the activation of PXR induced by rifampicin and the *EC_50_* values increased (**Table 1**).

**Table 1:** The effect of MTBP on PXR’s transcription factor activation.

Groups		MHCC97-H		MHCC97-L	
		control siRNA	siRNA of MTBP	empty vector	MTBP
*EC_50_* values of Rifampicin (μmol/L)	XREM-Luc	4.20 ± 0.33	–	4.91 ± 0.54	2.38 ± 0.85
	PXRE-Luc	5.13 ± 0.95	–	3.69 ± 0.71	1.88 ± 0.50
	DR3-Luc	6.22 ± 1.48	–	5.51 ± 0.65	2.64 ± 0.52
	ER6-Luc	5.40 ± 0.14	–	4.22 ± 0.69	2.85 ± 0.35
	*cyp3a4*	4.61 ± 0.20	–	5.50 ± 0.76	1.34 ± 0.84
	*abcb1*	5.08 ± 0.88	–	4.60 ± 0.16	2.70 ± 0.32

**Figure 2:**
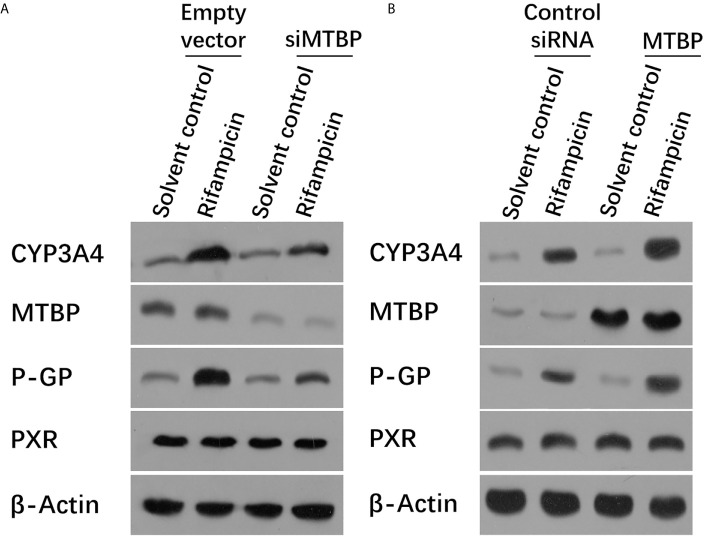
MTBP enhanced the expression of PXR downstream genes. **(A)** The MHCC97-H cells were transfected with a control siRNA or the siRNA of MTBP, whereas the MHCC97-L cells were transfected with an empty vector or MTBP **(B)**. Cells were treated with solvent control or rifampicin (10μmol/L concentration) and harvested for the western blot experiments. The protein levels of PXR, MTBP, CYP3A4 and P-gp (encoding by the acbc1) in these cells were examined by the antibodies. The β-Actin was chosen as the loading control.

Moreover, the overexpression of MTBP enhanced the mRNA or protein levels of PXR’s downstream genes, *cyp3a4* and *abcb1*, induced by rifampicin. The knockdown of MTBP decreased the mRNA and protein levels of PXR’s downstream genes, *cyp3a4* and *abcb1*, induced by rifampicin (**Table 1** and **Figure 2**). Therefore, MTBP enhanced the transcription factor activation of PXR.

### MTBP Interacted With PXR and Promoted the Accumulation of PXR in Nuclear and the Recruitment of PXR to the Promoter of Its Downstream Gene cyp3a4

**Figure 3:**
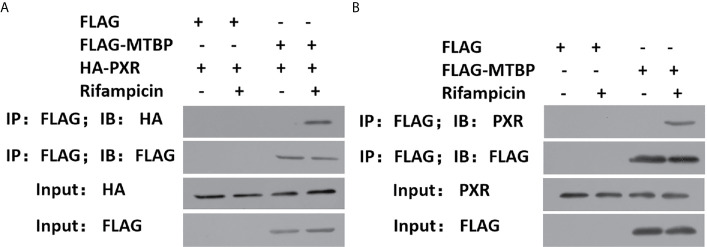
MTBP interacted with PXR. **(A)** The HEK293 cells that were co-transfected with HA-PXR or FLAG-MTBP were analyzed by co-IP experiments. **(B)** The MHCC97-H cells which were transfected with FLAG-MTBP were analyzed by co-IP experiments.

**Figure 4:**
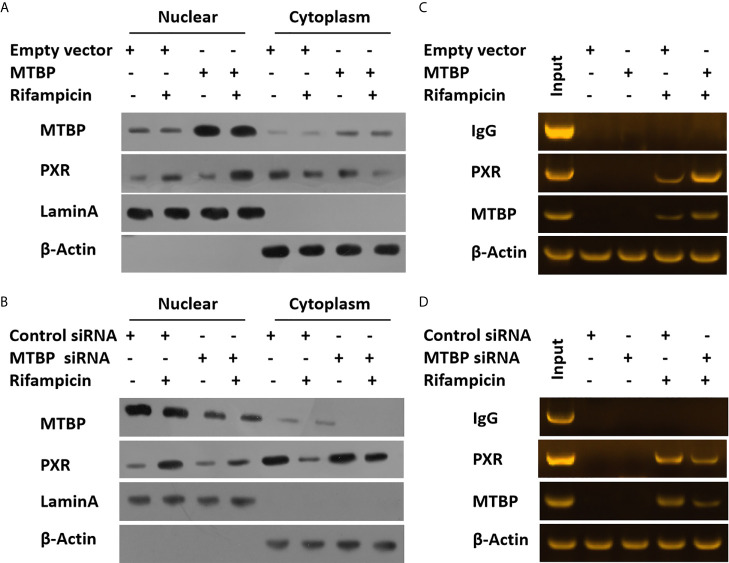
MTBP promotes the accumulation of PXR in nuclear or the recruitment of PXR to the promoter regions of its downstream gene. The MHCC97-L **(A)** cells were transfected with an empty vector or MTBP, whereas the MHCC97-H **(B)** cells were transfected with a control siRNA or the siRNA of MTBP. Cells were treated with a solvent control or rifampicin and harvested for the cellular sub-fraction experiments. The expression levels of PXR or MTBP in cellular sub-fractions was examined by the western blot assay. The Lamin A (the nuclear-skeletal protein) was used as the indicator of the nuclear sub-fraction, whereas β-Actin was used as the indicator of the cytoplasm sub-fraction. The MHCC97-L **(C)** cells were transfected with an empty vector or MTBP, whereas the MHCC97-H **(D)** cells were transfected with a control siRNA or the siRNA of MTBP. Cells were treated with a solvent control or rifampicin and harvested for the ChIP experiments.

To further examine the effect of MTBP on PXR, the protein interaction between MTBP and PXR was examined by co-immunoprecipitation (co-IP). As shown in **Figure 3**, MTBP interacted with PXR in MHCC97-H cells. Additionally, the overexpression of MTBP promoted the accumulation of PXR in nuclear induced by rifampicin, whereas the knockdown of MTBP deceased the accumulation of PXR in nuclear (**Figure 4**). The recruitment of PXR to the promoter of its downstream gene, *cyp3a4*, was examined by ChIP analysis (**Figure 4**). As shown in **Figure 4**, the overexpression of MTBP promoted the recruitment of PXR to the promoter region of *cyp3a4*. The knockdown of MTBP decreased the recruitment of PXR to the gene’s promoter region (**Figure 4**). Therefore, MTBP interacted with PXR and promoted the accumulation of PXR in nuclear and the recruitment of PXR to the promoter of its downstream gene.

### MTBP Accelerated the Eliminationof Sorafenib in HCC Cells

**Figure 5:**
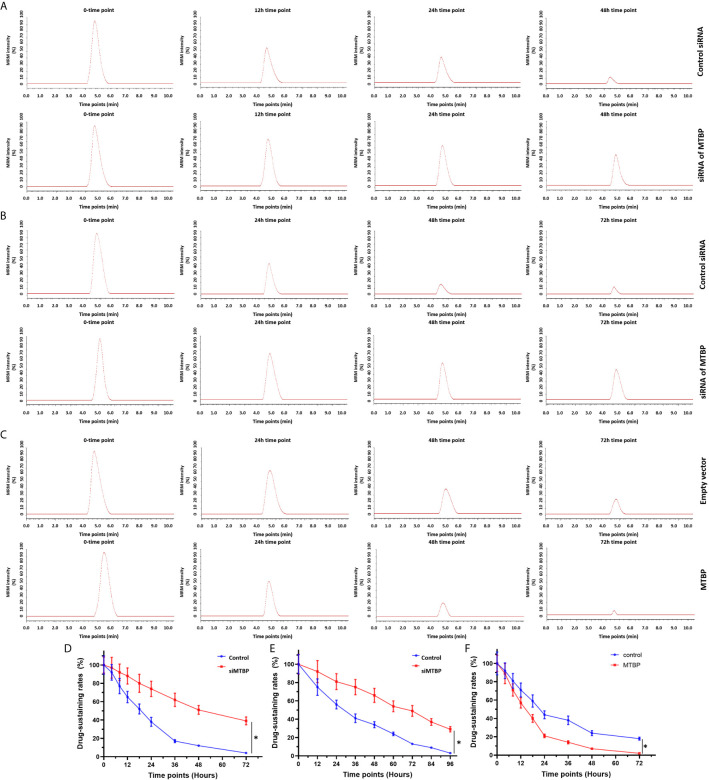
MTBP accelerates the clearance or metabolism of sorafenib in HCC cells. **(A, B)** The MHCC97-H **(A, B)** cells were transfected with a control siRNA or the siRNA of MTBP, whereas the MHCC97-L **(C)** cells were transfected with an empty vector or MTBP. The cells were cultured **(A, C)** and injected into nude mice to form subcutaneous tumor tissues. The sustaining of sorafenib in cultured HCC cells **(A, C)** or the subcutaneous tumors **(B)** was examined by the LC-MS/MS methods. The results were shown as the represented images of LC-MS/MS at represented time points or the drug sustaining curves **(D–F)**. *P < 0.05.

These results indicate that MTBP enhanced the activation of PXR and enhanced the expression of PXR’s downstream genes which mediated the metabolism of sorafenib. Therefore, the effects of MTBP on the elimination or clearance of sorafenib in HCC cells were examined by the LC-MS/MS methods. As shown in **Figure 5** and **Table 2**, the overexpression of MTBP in MHCC97-L cells accelerated the elimination of sorafenib in HCC cells and the subcutaneous tumor tissues, and the half-life time (t_1/2_ values) of sorafenib in HCC cells or tumor tissues was significantly reduced. The knockdown of MTBP in MHCC97-H cells accelerated the elimination of sorafenib in HCC cells or the subcutaneous tumor tissues, and the half-life time (t_1/2_ values) of sorafenib in HCC cells or tumor tissues was significantly reduced. Therefore, MTBP accelerated the elimination or clearance of sorafenib in HCC cells.

**Table 2:** The effect of MTBP on the metabolism or the clearance of sorafenib in HCC cells.

Groups		Half-life values (t_1/2_ values, hours)	
		Cultured Cells	Subcutaneous Tumors
MHCC97-H	control siRNA	19.30 ± 3.85	28.14 ± 2.81
	siRNA of MTBP	56.67 ± 5.58	71.27 ± 9.67
MHCC97-L	empty vector	22.95 ± 7.56	37.94 ± 7.23
	MTBP	13.81 ± 4.11	85.28 ± 6.52

### MTBP Enhanced the Resistance of HCC Cells to Molecular Targeting Agents

The antitumor effect of molecular targeting agents on HCC cells was examined by MTT experiments. As shown in **Supplemental Table 2**, sorafenib inhibited the survival of HCC cells in a dose-dependent manner. The overexpression of MTBP enhanced the resistance of MHCC97-L cells to sorafenib and the *IC_50_* values of sorafenib on MHCC97-L cells were increased (**Supplemental Table 2**). The knockdown of MTBP in MHCC97-H enhanced the antitumor effect of the drug on MHCC97-H cells and the *IC_50_* values of sorafenib on MHCC97-H cells decreased. Moreover, the overexpression of MTBP enhanced the resistance of MHCC97-L cells to molecular targeting agents regorafenib, lenvatinib, anlotinib and apatinib; the knockdown of MTBP enhanced the sensitivity of MHCC97-H cells to molecular targeting agents.

### MTBP Repressed the *In Vivo* Antitumor Effect of Sorafenib on HCC Cells’ Subcutaneous Growth and the Intrahepatic Growth

**Figure 6:**
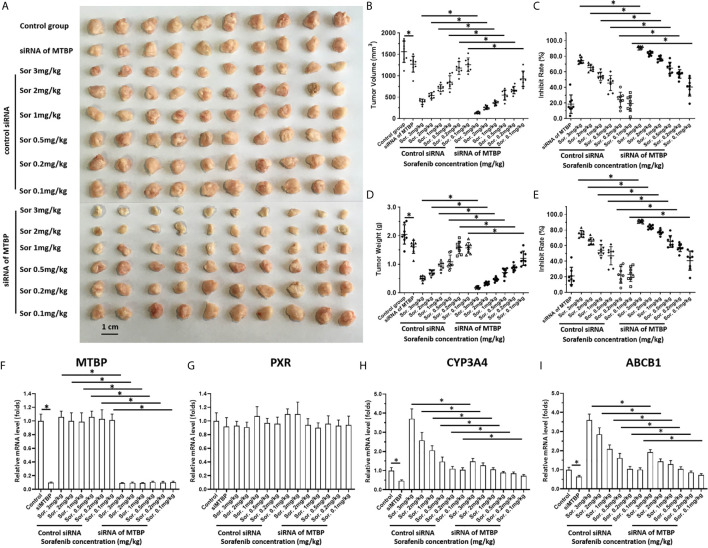
MTBP decreased the antitumor effect of sorafenib on the subcutaneous growth of HCC cells. MHCC97-H cells were cultured and transfected with a control siRNA or the siRNA of MTBP. Cells were injected into mice to form subcutaneous tumor tissues and the mice received sorafenib treatment *via* oral administration. The mice were harvested and the tumor tissues were collected. The results were shown as the images of tumor tissues **(A)**, tumor volumes **(B)**, inhibition rates of sorafenib calculated by tumor volumes **(C)**, tumor weights **(D)** or inhibition rates of sorafenib calculated by tumor weights **(E)**. **(F–I)** the expression level (the mRNA level) of MTBP **(F)**, PXR **(G)**, *cyp3a4* **(H)** or *abcb1* **(I)** in subcutaneous tumors **(A)** was examined by qPCR and shown as the scatter-plot images **(F–I)**. *P < 0.05.

To further examine the effect of MTBP on the *in vivo* antitumor activation of molecular targeting agents, the subcutaneous tumor model was used. As shown in **Figure 6** and **Table 2**, the molecular targeting agents inhibited the subcutaneous growth of HCC cells in nude mice. The overexpression of MTBP decreased the antitumor effect of molecular targeting agents and the *IC_50_* values increased (**Figure 6** and **Table 3**). The knockdown of MTBP enhanced the sensitivity of MHCC97-H cells to molecular targeting agents and the *IC_50_* values decreased (**Figure 6** and **Table 3**). The results were shown as the images of subcutaneous tumor tissue formed by MHCC97-H from the mice received sorafenib treatment and the *IC_50_* values of molecular targeting agents sorafenib, regorafenib, lenvatinib, anlotinib or apatinib (**Figure 6** and **Table 3**). The expression level of the related factors (MTBP, PXR, *abcb1* or *cyp3a4*) in the subcutaneous tumors (**Figure 6A**) was shown as **Figures 6F–I**.

**Table 3:** MTBP decreased the *in vivo* antitumor effect of molecular targeting agents.

Groups		*IC_50_* values (mg/kg)				
		Sorafenib	Regorafenib	Lenvatinib	Apatinib	Anlotinib
MHCC97-H	control siRNA	0.85 ± 0.37	0.90 ± 0.25	0.70 ± 0.10	1.35 ± 0.66	1.67 ± 0.78
	siRNA of MTBP	0.14 ± 0.02	0.25 ± 0.05	0.10 ± 0.01	0.38 ± 0.11	0.62 ± 0.23
MHCC97-L	empty vector	1.55 ± 0.30	1.31 ± 0.70	0.95 ± 0.44	2.10 ± 0.92	1.68 ± 0.50
	MTBP	–	–	–	–	–

**Figure 7:**
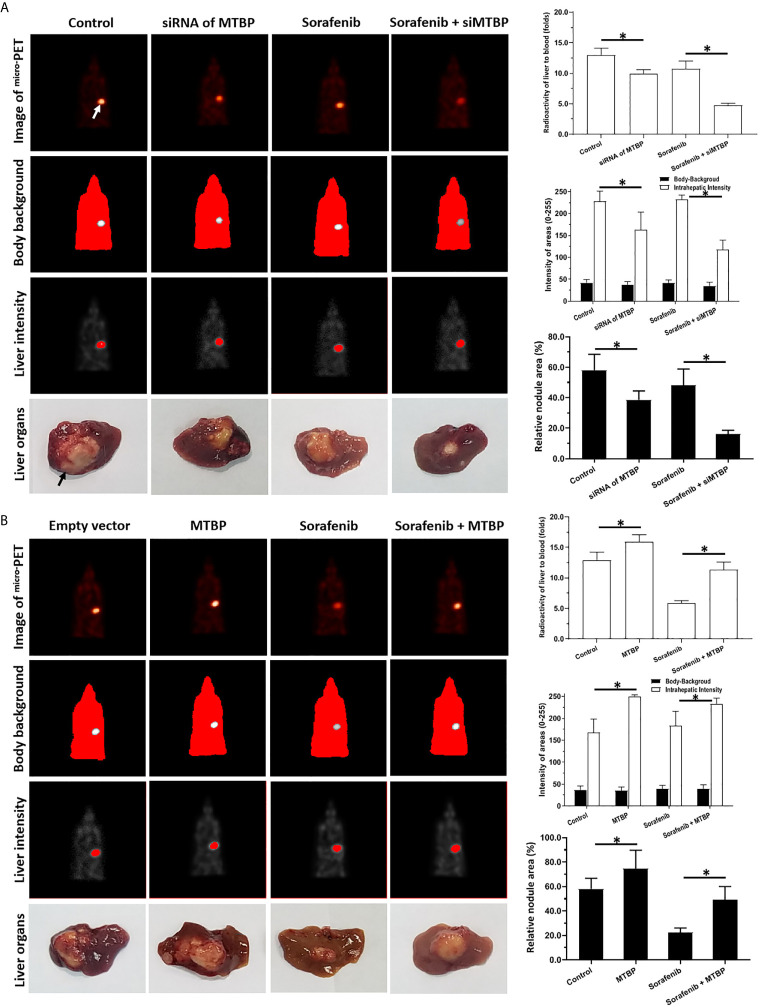
MTBP decreased the antitumor effect of sorafenib on the subcutaneous growth HCC cells. **(A)** MHCC97-H cells were cultured and transfected with a control siRNA or the siRNA of MTBP. Cells were first injected into mice to form subcutaneous tumor tissues; the micro-blocks of subcutaneous tissues were transplanted into the liver of each mouse to form intrahepatic tumor lesions (nodules). Then, the mice were given a sorafenib (0.5mg/kg) treatment *via* oral administration. The results were shown as the images of micro-PET, the quantitative results of micro-PET images **(A)** or the images of liver organs with nodules formed by HCC cells, the radio-activation of liver organs to blood, the intensity of liver regions to body-background (micro-PET’s images) or the relative area of the nodules. **(B)** MHCC97-L cells were cultured and transfected with empty vectors or the vectors of MTBP. Cells were first injected into mice to form subcutaneous tumor tissues and the micro-blocks of subcutaneous tissues were transplanted into the each liver to form the intrahepatic tumor lesions (nodules). Then, the mice were given sorafenib (3.0mg/kg) treatment *via* oral administration. The results were shown as the images of micro-PET, the quantitative results of micro-PET images **(A)** or the images of liver organs with nodules formed by HCC cells, the radio-activation of liver organs to blood, the intensity of liver regions to body-background (micro-PET’s images) or the relative area of nodules. *P < 0.05 The white arrow in **Figure 7** indicated the liver region from ^mirco^PET examination; whereas the black arrow in **Figure 7** indicated the nodules or lesions formed by HCC cells in the liver organs.

Next, the intrahepatic tumor models were used. As shown in **Figure 7**, the intrahepatic growth of HCC cells could form the intrahepatic nodules (tumor lesions) in the livers of the mice. The intrahepatic growth of HCC cells were shown as the micro-PET images (live imaging of small animals) and the images of livers with nodules (**Figure 7**). Treatment with sorafenib inhibited the intrahepatic growth of HCC cells in the liver of each of the mice (**Figure 7**). The overexpression of MTBP repressed the antitumor effect of sorafenib on both the micro-PET intensity images of the livers and the nodules formed by MHCC97-L (**Figure 7**). The knockdown of MTBP enhanced the antitumor effect of sorafenib both on the micro-PET intensity images of the livers and the nodules formed by MHCC97-H (**Figure 7**). Therefore, MTBP enhanced the resistance of HCC cells to molecular targeting agents.

### MTBP Is Associated With the Poor Prognosis of HCC Patients

**Figure 8:**
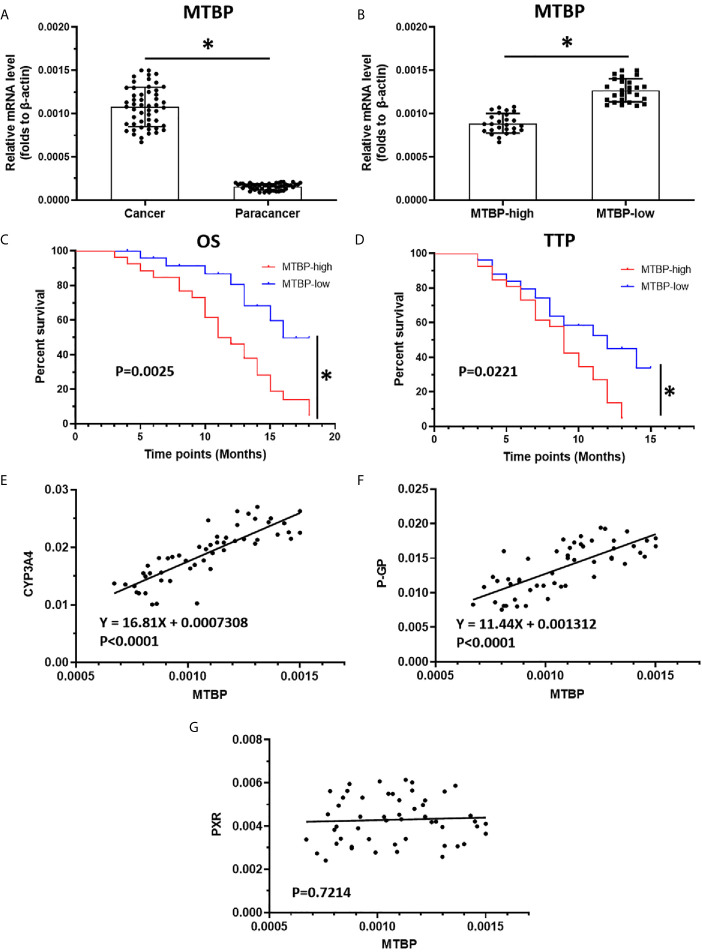
The high level of MTBP is related to the poor prognosis of advanced HCC patients who received sorafenib treatment. **(A)** The mRNA level of MTBP in the HCC clinical specimens and the paired non-tumor specimens were examined by qPCR. **(B)** The patients with advanced HCC were divided into two groups (MTBP-high or MTBP-low) according to the median value of MTBP’s mRNA level. **(C, D)** The OS or TTP of MTBP-high or MTBP-low groups’ patients are shown as survival curves. **(E, F)** The relationship between the expression of MTBP and the expression of PXR’s downstream genes CYP3A4 **(E)** and ABCB1 is represented **(F)**. **(G)** The co-relation between MTBP with PXR in HCC clinical specimens and was shown as scatter-plot images. *P < 0.05.

The above results indicate that MTBP could interact with PXR and enhance the transcription activation of PXR to mediate the resistance of HCC cells to sorafenib. To confirm the specificity of MTBP and the clinical significance of MTBP, the expression of MTBP in advanced HCC clinical specimens was examined and the patients were divided into two groups (MTBP-high group and MTBP-low group) according to the median value of the endogenous MTBP level (**Figures 8A–D**). As shown in **Figures 8C**, **D** and **Table 4**, the prognosis for patients in MTBP-high groups is much worse than that of patients in MTBP-low groups (the TTP [time to progress] is 9.0 months [median], 95% CI [7.8-10.2 months] versus 12.0 months [median], 95% CI [9.0-12.6 months], log-rank P=0.022; the OS [overall survival] is 11.0 months [median], 95% CI [9.9-13.4 months] versus 16.0 months [median], 95% CI [3.5-16.8 months], log-rank P=0.003).

**Table 4:** MTBP expression and clinical outcome of patients received sorafenib treatment.

	MTBP mRNA expression		P
	High (n = 26)	Low (n = 26)	
TTP	9.0	12.0	0.022
	7.8-10.2 (M)	9.0-12.6 (M)	
OS	11.0	16.0	0.003
	9.9-13.4 (M)	13.5-16.8 (M)	

TTP, time to progress; OS, overall survival; M, months.

Moreover, the relationship between the expression of MTBP with the downstream genes of PXR, *cyp3a4* and *ABCB1* in clinical specimens was examined. As shown in **Figures 8E, F**, the expression of MTBP-1 was positively related to the expression of *cyp3a4* (P<0.0001) and *ABCB1* (P<0.0001) in clinical specimens. As expectative, the expression of MTBP in HCC specimens did not associate with PXR (**Figure 8G**). Therefore, the data from the clinical specimens confirmed MTBP’s pro-proliferative and oncogenic activation and the effect of MTBP on PXR’s activation.

### The Specificity of MTBP on PXR’s Activation

Further experiments were performed in Huh-7 cells (P53-deficient HCC cells), HEK293 (PXR-negative cell line), and using PXR antagonist ketoconazole treating cells. The activation of PXR was revealed by the *EC_50_* values of rifampicin on luciferase reporters or mRNA level of PXR’s downstream genes. As shown in **Supplemental Table 3**, overexpression of MTBP enhanced the activation of luciferase reporters or the mRNA level of PXR’s downstream genes, *cyp3a4* or *abcb1*; knockdown of MTBP decreased the transcription factor activation of PXR. Treatment of ketoconazole almost blocked the activation of PXR. Moreover, overexpression of MTBP could only enhanced the activation of luciferase reporters or the mRNA level of PXR’s downstream genes, *cyp3a4* or *abcb1* in the presence of PXR (**Supplemental Table 4**). Therefore, The specificity of MTBP on PXR’s activation was confirmed by using the multi-assays.

## Discussion

The molecular targeting agents represented by sorafenib are of great significance for the treatment of advanced HCC but it has been clearly reported that patients’ sensitivity to the drug varies according to each individual. During the treatment, patients are prone to developing resistance to molecular targeting agents (33). Some cellular signaling pathways, including CSCs (cancer stem cells), the Notch pathway, the EMT (epithelial-mesenchymal transition) and mTOR (mammalian target Rapamycin) can affect the antitumor activity of molecular targeting agents (34–40). Results from recent studies have shown that metabolic factors are also important mechanisms for the resistance of various molecular targeted therapies. Yin et al. reported that the use of the SREBP-1 inhibitor Betulin down-regulated the transcription factor activity of SREBP-1 to increase the sensitivity of HCC cells to sorafenib (23). Ma et al. reported that miR-6077 can promote the antitumor effect of anlotinib on NSCLC (non-small cell lung cancer) cells by down-regulating the expression level of GLUT-1 (glucose transporter 1) sensitivity (41). SREBP-1 (Sterol Regulatory Element Binding Protein-1) and GLUT-1 are important regulators of cellular material and energy metabolism (42–45) for modulating the microenvironment of tumor cells. Therefore, it is necessary to further examine the participation of the aberrant metabolism of malignant tumor cells in the progress or resistance of cancer cells to antitumor agents (46).

PXR can act as a regulatory hub for the elimination and detoxification of exogenous drugs and toxicants through its downstream resistance-related genes, known to induce the metabolism and elimination of molecularly-targeted drugs, resulting in the resistance of HCC cells to the drugs (47, 48). In this study, MTBP interacted with PXR and enhanced the transcription factor activation of PXR which eventually caused the accelerated elimination or clearance of sorafenib. These results not only extend our knowledge about PXR’s regulation but also our knowledge about resistance to the drug.

MTBP was first identified by an MDM2-interacting protein (49). Recently, multiple points of evidence have indicated the pro-proliferative and oncogenic activation of MTBP. This activation has been carried out by multiple mechanisms: (1) MTBP is co-amplified with c-MYC and functions as c-Myc’s co-activator (50); (2) MTBP could enhance the activation of ZEB2 to induce the EMT process of cancerous cells (13); (3) MTBP inhibits the apoptosis of cancer cells *via* suppression of the MDM2/P53 axis (14); and (4) MTBP can enhance the metastasis of HCC cells *via* the MDM2-Mediated E-Cadherin Degradation (11).

In this study, MTBP interacted with PXR and functioned as the co-activator of PXR to enhance the resistance of HCC cells to molecular targeting agents. Our results are consistent with those of our previous publication in which we mentioned that MTBP can function as an activator of ZEB or c-MYC (50). ZEB is the important transcription factor mediating the EMT process of human cancer cells; c-MYC not only promotes the proliferation of cancer cells, it also participates in the aberrant metabolism of cancer cells (51–55). Therefore, in the future it would be valuable to examine the interaction between MTBP and the transcription factors. MTBP has also been found to act as a tumor suppressor in some cases. Findings from our previous work and related data indicate that MTBP may not significantly affect the proliferation of HCC cells but it does inhibit the metastasis of HCC cells by inhibiting the activation of ACTN (alpha-actinin 4) (56–59). It is possible that ACTN is the main mechanism by which MTBP functions and the different expressions of ACTN, c-MYC and ZEB in different cells may affect the role of MTBP (56–59).

In this study we not only detected the expression of the drug-resistant genes CYP3A4 and ABCB1, we also directly observed the effects of MTBP on the metabolism and clearance of the molecularly-targeted drug sorafenib. The overexpression of MTBP can accelerate the metabolism and clearance of sorafenib in HCC cells and tissues while the knocking down of MTBP expression can prolong the retention time of sorafenib in HCC cells and tissues. Because the drug has been used in clinical treatments for a long time, its drug metabolism-related characteristics, such as those noted in our previous results, have established an LC-MS/MS method for detecting it. The application time of several other new molecules is shorter than that of sorafenib and data related to LC-MS/MS methodologies for these drugs is rarely reported. It will be valuable to establish these methods in the future for detecting the effects of MTBP on the metabolism and clearance of regorafenib, lenvatinib, apatinib and anlotinib in HCC cells.

## Data Availability Statement

The original contributions presented in the study are included in the article/**Supplementary Material**, further inquiries can be directed to the corresponding authors.

## Ethics Statement

The studies involving human participants were reviewed and approved by The Fifth Medical Center of Chinese PLA General Hospital. The patients/participants provided their written informed consent to participate in this study. The animal study was reviewed and approved by The Fifth Medical Center of Chinese PLA General Hospital. Written informed consent was obtained from the owners for the participation of their animals in this study.

## Author Contributions 

QB, JH, and B-aL. conceived the main ideas and wrote the paper. JJH and JC supervised the study. QJ and YM developed major methodologies, databases, reagents, and primary experiments. XM, LS, JH, and B-aL. analyzed different aspects of the results. All authors contributed to the article and approved the submitted version.

## Conflict of Interest

The authors declare that the research was conducted in the absence of any commercial or financial relationships that could be construed as a potential conflict of interest.
