# Supplementary material for: MDM2 Binding Protein Induces the Resistance of Hepatocellular Carcinoma Cells to Molecular Targeting Agents via Enhancing the Transcription Factor Activity of the Pregnane X Receptor
Source: Front Oncol. 2021 Jun 24;11:715193. doi: 10.3389/fonc.2021.715193 (PMC8264664; doi:10.3389/fonc.2021.715193)
Supplement: Supplementary file 1 [file DataSheet_1.zip › Supplementary Table 1.DOC]

Supplemental Table 1 The weights of micro-block (mg) in the intrahepatic experiments

| Nodule number | | No. 1 | No. 2 | No. 3 | No. 4 | No. 5 | No. 6 | No. 7 | No. 8 | No. 9 | No. 10 |
| --- | --- | --- | --- | --- | --- | --- | --- | --- | --- | --- | --- |
|  |  | weights of micro-block (mg) | | | | | | | | | |
| MHCC97-H | control | 1.8 | 1.7 | 2.0 | 2.2 | 1.5 | 2.3 | 2.2 | 1.9 | 1.8 | 2.4 |
|  | siMTBP | 2.3 | 1.9 | 2.1 | 1.8 | 1.7 | 1.5 | 2.6 | 2.0 | 1.8 | 2.1 |
|  | Sorafenib | 2.1 | 2.3 | 1.7 | 1.9 | 2.2 | 2.3 | 1.7 | 1.8 | 1.9 | 1.7 |
|  | Sorafenib + siMTBP | 1.5 | 2.3 | 1.8 | 1.9 | 1.6 | 2.3 | 2.2 | 2.2 | 2.0 | 1.9 |
| MHCC97-L | empty vector | 1.8 | 1.9 | 2.4 | 1.6 | 2.5 | 2.1 | 1.8 | 2.1 | 1.7 | 1.9 |
|  | MTBP | 2.0 | 1.6 | 2.4 | 1.8 | 2.2 | 2.3 | 1.7 | 2.2 | 1.6 | 1.8 |
|  | Sorafenib | 1.7 | 2.2 | 1.9 | 1.8 | 1.8 | 2.1 | 2.2 | 1.9 | 2.1 | 1.8 |
|  | Sorafenib + MTBP | 2.2 | 1.9 | 1.8 | 1.6 | 1.7 | 2.3 | 1.8 | 1.5 | 2.2 | 2.5 |
