# Supplementary material for: MDM2 Binding Protein Induces the Resistance of Hepatocellular Carcinoma Cells to Molecular Targeting Agents via Enhancing the Transcription Factor Activity of the Pregnane X Receptor
Source: Front Oncol. 2021 Jun 24;11:715193. doi: 10.3389/fonc.2021.715193 (PMC8264664; doi:10.3389/fonc.2021.715193)
Supplement: Supplementary file 1 [file DataSheet_1.zip › Supplementary Table 2.DOC]

Supplemental Table 2 MTBP enhanced the resistance of HCC cells to molecular targeting agents

| Groups | | *IC_50_* values (μmol/L) | | | | |
| --- | --- | --- | --- | --- | --- | --- |
|  |  | Sorafenib | Regorafenib | Lenvatinib | Apatinib | Anlotinib |
| MHCC97-H | control siRNA | 1.43±0.68 | 1.24±0.59 | 0.63±0.26 | 1.77±0.55 | 2.83±0.68 |
|  | siRNA of MTBP | 0.21±0.02 | 0.25±0.05 | 0.10±0.01 | 0.44±0.20 | 0.56±0.15 |
| MHCC97-L | empty vector | 2.41±0.62 | 2.52±0.78 | 1.61±0.53 | 2.70±0.82 | 3.84±0.45 |
|  | MTBP | - | - | - | - | - |
