# Supplementary material for: MDM2 Binding Protein Induces the Resistance of Hepatocellular Carcinoma Cells to Molecular Targeting Agents via Enhancing the Transcription Factor Activity of the Pregnane X Receptor
Source: Front Oncol. 2021 Jun 24;11:715193. doi: 10.3389/fonc.2021.715193 (PMC8264664; doi:10.3389/fonc.2021.715193)
Supplement: Supplementary file 1 [file DataSheet_1.zip › Supplementary Table 3.DOC]

Supplemental Table 3. The effect of MTBP on PXR’s activation in Hu-7 cells

| Groups | control | MPBP | siMTBP | MTBP + Ketoconazole |
| --- | --- | --- | --- | --- |
|  | *EC_50_* values on PXR's activation (μmol/L) | | | |
| XREM-Luc | 6.82±0.67 | 1.75±0.18 | 9.03±1.01 | - |
| PXRE-Luc | 7.36±0.87 | 3.55±0.10 | - | - |
| DR3-Luc | 6.91±0.39 | 2.54±0.70 | - | - |
| ER6-Luc | 7.75±0.33 | 2.21±0.41 | 8.57±0.86 | - |
| cyp3a4 | 5.35±0.67 | 1.19±0.17 | - | - |
| abcb1 | 6.84±0.11 | 4.23±0.28 | - | - |
