# Supplementary material for: MDM2 Binding Protein Induces the Resistance of Hepatocellular Carcinoma Cells to Molecular Targeting Agents via Enhancing the Transcription Factor Activity of the Pregnane X Receptor
Source: Front Oncol. 2021 Jun 24;11:715193. doi: 10.3389/fonc.2021.715193 (PMC8264664; doi:10.3389/fonc.2021.715193)
Supplement: Supplementary file 1 [file DataSheet_1.zip › Supplementary Table 4.DOC]

Supplemental Table 4. The effect of MTBP on PXR’s activation in HEK293 cells

| Groups | control | MTBP | PXR | MPBP + PXR | MTBP + Ketoconazole + PXR |
| --- | --- | --- | --- | --- | --- |
|  | *EC_50_* values on PXR's activation (μmol/L) | | | | |
| XREM-Luc | - | - | 5.22±0.74 | 1.84±0.33 | - |
| PXRE-Luc | - | - | 7.02±0.88 | 1.58±0.13 | - |
| DR3-Luc | - | - | 5.423065 | 2.78±0.60 | - |
| ER6-Luc | - | - | 6.67±0.59 | 2.66±0.32 | - |
